# Role of Megafauna and Frozen Soil in the Atmospheric CH_4_ Dynamics

**DOI:** 10.1371/journal.pone.0093331

**Published:** 2014-04-02

**Authors:** Sergey Zimov, Nikita Zimov

**Affiliations:** Northeast Science Station, Pacific Institute for Geography, Russian Academy of Sciences, Cherskii, Russia; DOE Pacific Northwest National Laboratory, United States of America

## Abstract

Modern wetlands are the world’s strongest methane source. But what was the role of this source in the past? An analysis of global ^14^C data for basal peat combined with modelling of wetland succession allowed us to reconstruct the dynamics of global wetland methane emission through time. These data show that the rise of atmospheric methane concentrations during the Pleistocene-Holocene transition was not connected with wetland expansion, but rather started substantially later, only 9 thousand years ago. Additionally, wetland expansion took place against the background of a decline in atmospheric methane concentration. The isotopic composition of methane varies according to source. Owing to ice sheet drilling programs past dynamics of atmospheric methane isotopic composition is now known. For example over the course of Pleistocene-Holocene transition atmospheric methane became depleted in the deuterium isotope, which indicated that the rise in methane concentrations was not connected with activation of the deuterium-rich gas clathrates. Modelling of the budget of the atmospheric methane and its isotopic composition allowed us to reconstruct the dynamics of all main methane sources. For the late Pleistocene, the largest methane source was megaherbivores, whose total biomass is estimated to have exceeded that of present-day humans and domestic animals. This corresponds with our independent estimates of herbivore density on the pastures of the late Pleistocene based on herbivore skeleton density in the permafrost. During deglaciation, the largest methane emissions originated from degrading frozen soils of the mammoth steppe biome. Methane from this source is unique, as it is depleted of all isotopes. We estimated that over the entire course of deglaciation (15,000 to 6,000 year before present), soils of the mammoth steppe released 300–550 Pg (10^15^ g) of methane. From current study we conclude that the Late Quaternary Extinction significantly affected the global methane cycle.

## Introduction

**Figure 1:**
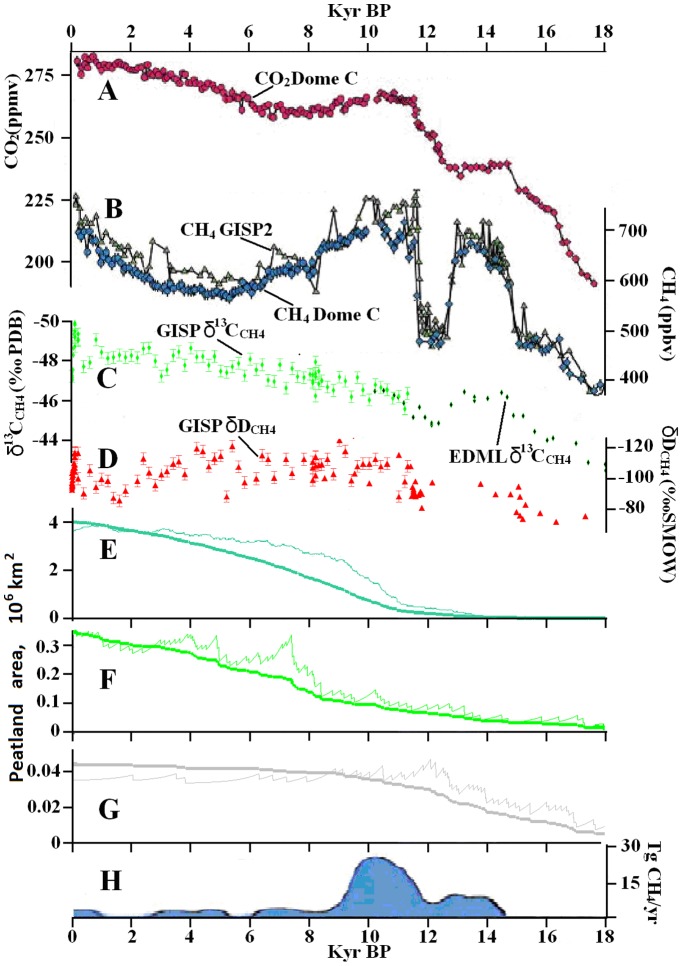
The dynamics of atmospheric: A) CO_2_ [17]; B) CH_4_ [17]; C) δ^13^CH_4_ [12], [13]; and D) δDCH_4_ [11], [13]. GISP1&2–Greenland Ice Sheet Projects. EDML - EPICA Dronning Maud Land Ice Core. The dynamics of peatland areas: E) boreal [17], [18]; F) tropical [18]; and G) southern [18]. Thin lines represent emissions from these wetlands based on the model of wetland succession (arbitrary unit). H) Methane emissions from northern Siberian permafrost [8].

Ice core analyses indicate that during the Pleistocene-Holocene transition (18,000 to 11,000 year before present (BP)), coincident with a rise in Greenland’s temperatures, atmospheric methane content increased from ∼1000 Tg (1 Tg = 10^12^ g) to ∼2000 Tg (Fig. 1A) [1]–[5]. The modern atmospheric lifetime of methane is approximately 10 years [6] (atmospheric methane lifetime is the ratio between atmospheric methane content and global annual methane flux). If the hypothesis that oxidation rates of methane in the atmosphere did not vary substantially in the past is accepted, and the lifetime of methane in the atmosphere stayed stable, then global CH_4_ emissions during Pleistocene-Holocene transition (18–11 ka BP) increased from ∼100 Tg/yr to ∼200 Tg/yr. In the Holocene, relative to the late Pleistocene, the climate was stable while global methane emission was not (Fig. 1B). From these data, it can be assumed that during 15–6 ka BP strong methane sources existed. By 6 ka BP, these sources vanished or substantially decreased in strength. This, in turn, caused a decrease in atmospheric concentrations of methane. Later, in the second part of the Holocene, other sources appeared (or were activated) and global emissions of methane rose by approximately 50 Tg/yr.

A comparison of ice core data from Greenland and Antarctica indicated that in the Last Glacial Maximum (LGM), atmospheric methane concentrations were roughly equal in the southern and northern hemispheres (Fig. 1B) [5]. Which indicate approximately equal methane production in both northern and southern hemisphere. However, simultaneous with a CH_4_ concentration rise, an interhemispheric CH_4_ gradient appeared, indicating the arrival of a strong northern source of methane. Based on this gradient, a northern methane source of 40–50 Tg/yr for the Bølling-Allerod (∼14.5–13 ka BP), 40 Tg/yr for the Younger Dryas (∼13–11.8 ka BP), and 60–70 Tg/yr for the Preboreal (∼11.8–9 ka BP) was determined [2]–[5], [8]. In contrast to the atmospheric methane concentration, the interhemispheric gradient stayed relatively stable over the course of the Holocene.

The origins of the dynamics noted above are actively debated. The rise in atmospheric methane during Pleistocene-Holocene transition can be explained by the expansion of boreal or tropical wetlands [1], the destabilization of gas clathrates [7], or the production of CH_4_ following the thawing of organic rich permafrost under anaerobic conditions [8].

Between 5 and 1 ka BP in a stable climate, methane emissions increased by at least 50 Tg/yr (Fig. 1B). The reason for this phenomenon is unclear to us. We are not convinced by the hypothesis that humans early farming have caused this rise [9]. Modern rice paddies and livestock produce 70 and 90 Tg CH_4_/yr respectively [6].During 5 to 1 ka BP, the human population was two orders of magnitude lower than at present [10], therefore we think that increase in human population is not a sufficient explanation for the rise. Additionally, the first rice paddies were cultivated in areas of natural wetlands, and domestic animals replaced wild animals in pastures.

**Figure 2:**
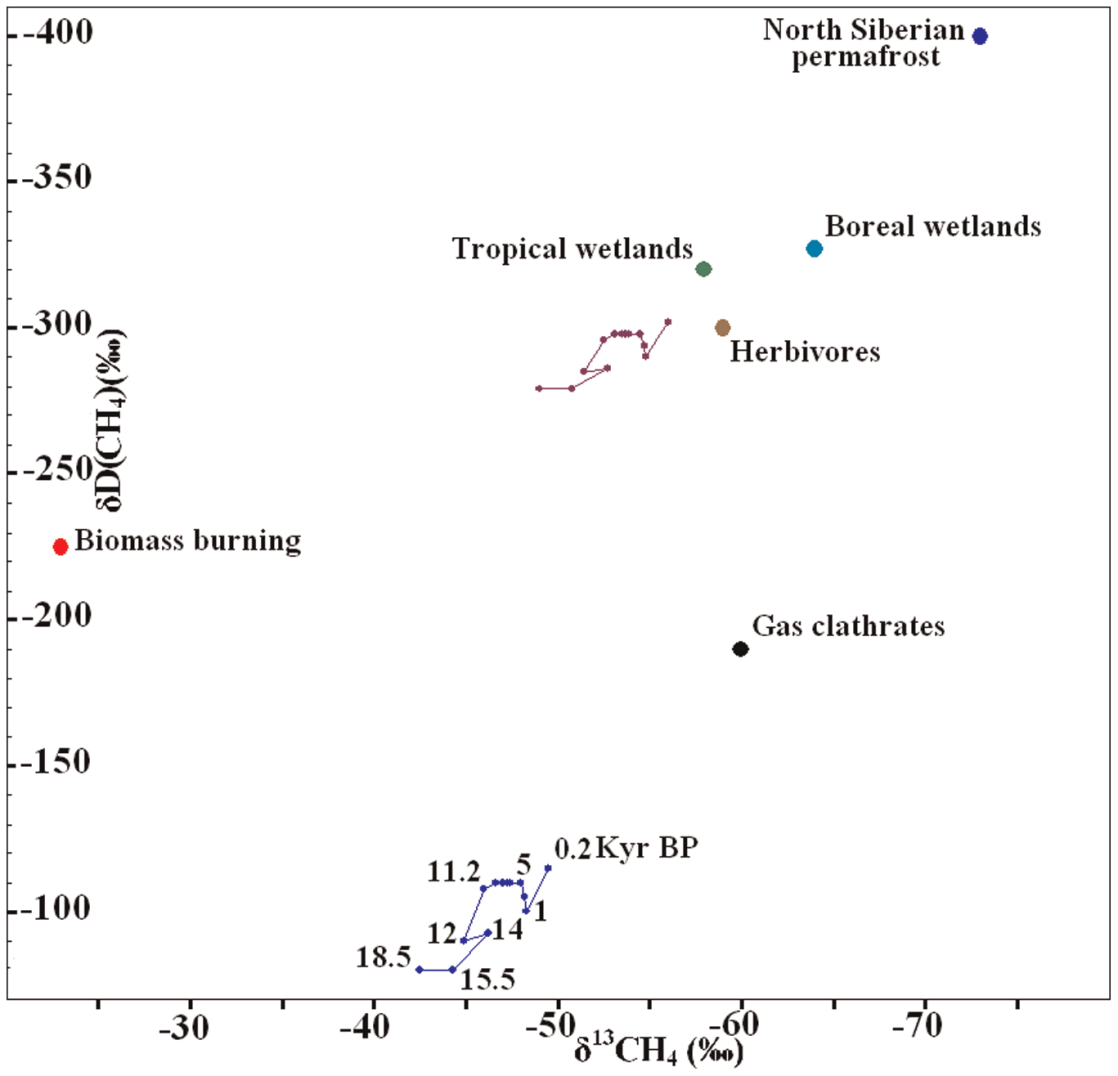
The isotopic signatures (^13^C and deuterium) of primary natural sources [12]. The blue line represents the dynamics of the isotopic content of methane in the atmosphere above Greenland from the LGM to the pre-industrial [11]–[13]; and purple line represents the dynamic of the isotopic content of the weighted average source calculated for conditions as in the scenario in Fig. 3A.

The isotopic composition of atmospheric methane obtained from ice core analyses allows us to understand the dynamics of primary methane sources [6], [11], [12]. Methane from different sources is different in isotopic composition (Fig. 2). The dynamic of isotopic methane signature from the LGM to the present is now known (Fig. 1C and 1D) [11]–[13]. This allows evaluation of the input to global methane emissions from different sources.

An attempt to estimate primary CH_4_ sources during Pleistocene-Holocene transition was made by *Fischer et al.* [12]. The authors employed a model that describes the dynamics of atmospheric methane, its interhemispheric gradient, and the ^13^C and deuterium budgets. However Monte Carlo approaches allowed the dynamics for only two sources to be calculated and according to their results, biomass burning emissions in the LGM for the 3.7 year atmospheric CH_4_ lifetime were 65 Tg/yr, and for the 5.6 year lifetime equalled 41 Tg/yr (the same as the biomass burning emissions of today [6]). Boreal wetland emissions during the LGM were 4 Tg/yr and later increased to 54 Tg/yr in the Preboreal, regardless of lifetime duration [12]. The boreal wetland dynamic was predictable, since in the model the presence of an interhemispheric gradient was fully dependent on the presence of a single northern source – boreal wetlands (permafrost was not considered). The Fischer model assumed that interhemispheric gradient in the LGM was small and, therefore the model showed that boreal wetland emissions were small, as well. Over the course of Pleistocene-Holocene transition interhemispheric gradient substantially increased and therefore, the model was supposed to show and did show a strong rise in boreal wetland emissions regardless of the dynamic of the isotopic content of atmospheric methane and its lifetime.

Below, we present our isotopic model. The general idea and equations of the model are similar to the Fischer model and most input parameters are the same. However, the following differences between our and Fischer’s models allowed us to constrain or estimate all main methane sources from the LGM to the present: 1) we obtained measurements not only for the Pleistocene-Holocene transition but also for the Holocene; 2) we added into the model an additional northern source – permafrost; 3) we did not add restrictions connected with the interhemispheric gradient; interhemispheric methane gradient dynamics data was utilized only to test the results of our modelling: we checked by modelling whether the strengths of the northern sources corresponded with the values of the interhemispheric gradient; 4) we set emissions dynamics for boreal and tropical wetlands using a model based on a large global database of basal peat initiation dates. These allowed us to reduce the number of unknowns in the isotopic model and, in contrast to Fischer’s results, allowed us to calculate our system of equations, not with the Monte Carlo approach, but analytically.

## Background

Before analysing the isotopic model, we want to discuss current knowledge about the glacial–interglacial dynamic of the main methane sources. Below we compare the results obtained from the modelling with these initial estimates.

### Gas Clathrates

The storage of gas clathrates in the bottom sediments of seas and oceans is approximately 10000–20000 Pg [14]. But we doubt that this source seriously influenced the methane concentration in the atmosphere. Our observations of methane bubbling in different bottom sediments [15], have indicated that if CH_4_ production in bottom sediments is low, bubbling occurs only at low pressure. In lakes, bubbling occurs at record low atmospheric pressure. In rivers, channels, and on the shelf of the East-Siberian Sea it occurs at low water levels. Record low pressure in the sediments occurs rarely and during that short time period releases the methane which had accumulated in the sediments for several months or even years. As soon as the pressure (water level) increases, bubbling immediately stops [15]. We assume that the same processes are correct for gas clathrates on the continental slope and the ocean floor. During deep glacial ocean regression the pressure in the bottom sediments declined much more strongly than in lakes and rivers. Gas clathrates emissions during the regression should have increased, with the maximum emissions being at the deepest regression, and after the LGM peak, the emissions should have immediately stopped as soon as ocean levels began to rise. However, for atmospheric methane dynamics we did not determine such an outcome. Gas clathrate methane has a very high concentration of deuterium isotope [11], [12] and, therefore, even minor changes in the emissions of this source into the atmosphere, should substantially change the deuterium content in the atmospheric methane; however, before and after the LGM, the deuterium content in the atmosphere remained stable (Fig. 1C) [11].

Gas clathrate destabilization during ocean regression must have taken place [14]. But this methane did not penetrate to the atmosphere. Field experiments indicate that methane bubbles of a few centimetres in diameter lose ∼20% of their methane content per 10 m of updrift (methane within the bubble is replaced by nitrogen and oxygen) [15]. Therefore, methane from the sea sediments (from hundreds of metres in depth and more) was dissolved in the ocean and oxidized to CO_2_ [16]. During Pleistocene-Holocene transition the deuterium content of atmospheric CH_4_ decreased (Fig. 1C). Amongst natural sources, the δDCH_4_ of gas clathrates is the heaviest (Fig. 2). Therefore, the destabilization of gas clathrates could not be the cause of an atmospheric CH_4_ rise [11], since a source with low deuterium content is required [11].

### Wetlands

Wetland CH_4_ emission dynamics can be reconstructed using the dynamics of peat accumulation [17], [18]. The conditions required for methanogenesis and peat accumulation are similar – plant remains should appear under anaerobic conditions (i.e., isolated by a layer of water). Some wetlands become episodically covered with water. In such places peat does not accumulate, but methane is still occasionally emitted. The conditions for the existence of peatlands and episodic wetlands are similar – the more humid the climate and the less the drainage, the wetter the soils. If, in a specific region due to climate change, the area of peatlands increases, then the area of episodic wetlands will increase as well. Therefore, the dynamics of wetland areas as well as their CH_4_ emissions should be correlated with the dynamics of peatland areas.

The dynamics of peatland areas for three climatic zones are shown in Figures 1E, 1F and 1G (thick lines). These graphs were built based on 1,700 ^14^C basal peat initiation dates [17], [18]. This is the world’s biggest global database of peat initiation dates and it can allow reconstruction of the world wetland dynamics. From these graphs it can be concluded that during Pleistocene-Holocene transition, tropical and boreal peatlands were rare and also the rates of peat accumulation were an order of magnitude slower than during the stages of active peatland expansion [18]. Basal peat dates have not been determined for the LGM, but there are peatlands older than the LGM [17], [18]. This means that in the LGM there were few peatlands.

There is no other evidences of abundancy of wetlands during the LGM [19]. An analysis of paleo-vegetation maps indicates that, during the LGM, forested areas were ten times smaller than during the Holocene [19], [20]. Sphagnum spores (a reliable proxy for moist conditions) are abundant during the Holocene, but they are absent in pollen spectra during the LGM [20], [21]. For northern territories with underlying permafrost, another reliable indicator of anaerobic conditions is soil methane [22]. In the frozen soils of the Holocene and other interglacial periods, methane is frequently present [22]. In soils that experience thawing, methane is always abundant, while in all of the frozen soils that accumulated during the LGM it is absent [22]. One of the additional reasons for glacial landscape dryness is a lowering of the erosion basis. At ocean regression in the Pleistocene, river mouths lowered, inclinations of the rivers increased and river beds deepened. Following the erosion basis, drainage of the territory increased and the area of floodplains reduced. Most of the current vast moist plains in the glaciation were well drained uplands, and only when deglaciation finished and the water level (ground water level) reached the modern level, did landscape saturation occur. The Holocene climate was relatively stable but because of slow processes like land grading, silting of river beds and depressions, the area of peatlands increased over the course of Holocene. Accounting for all the above, we accept that wetlands during the LGM were rare. Now, knowing the dynamic of the wetlands area, we will try to reconstruct the methane emission dynamic from the wetlands.

The work of *McDonald et al.* [17] noted that, during the initial stage of peatland formation (high productive grass-herb formation), CH_4_ emissions were higher than during later low productive stages. Therefore, for reconstructing peatland methane emissions, we utilized a model in order to take peatland succession into consideration. In the model we accept that maximum methane emission occurs at the time of peatland initiation, and declines afterwards. We assumed various scenarios of successions and in all cases the peatland succession model indicates that methane emission graph differ slightly from the dynamics graph of the peatland area. Only by accepting a very long stage of succession are the differences visible. In Figures 1E, 1F, and 1G the thin lines were built by assuming that, at the moment of initiation of every peatland, methane production was at a maximum, and very slowly declined. In 600 years, emissions were halved; in 1,600 years they were a quarter of their initial level, and six times lower during late succession. Even in the cases of long and deep succession, differences in graph shapes were not that pronounced.

Peat is a very good proxy for anaerobic conditions [17], [18]. It is likely that the graphs in Figures 1D–F are the most precise data reflecting the dynamics of methane from wetlands. These graphs reflect the dynamic of methane emission in arbitrary units, but if we know the modern (pre-industrial) emission of boreal and tropical wetlands then, based on these graphs, we can estimate wetland methane emissions for any other time slice (up to the LGM). Utilizing these data we can determine whether wetlands could be the reason for the methane rise during Pleistocene-Holocene transition.

Area of southern wetlands (mostly Patagonian) smoothly grew from 18 to 12 ka BP (Fig. 1G), but their area was ∼1% of the modern boreal wetland area. Therefore, it is unlikely they had an effect on the global dynamic of atmospheric methane. One can clearly see that the strong expansion of boreal wetlands only began around 11 ka BP, three thousand years after the methane atmospheric concentration reached Holocene levels and a strong interhemispheric gradient appeared (Fig. 1B). Tropical wetland expansion took place at an even later date. From these dynamics we can conclude that both tropical and boreal wetlands had little influence on the methane concentration rise during Pleistocene-Holocene transition. Between 11 and 7 ka BP numerous young wetlands appeared worldwide (Figs. 1D–F), so maximum wetland emissions should be expected during this period of time. However, atmospheric methane concentrations did not rise and instead declined (Fig. 1B), indicating that Pleistocene-Holocene transition other stronger sources existed that sharply declined in strength from 11 to 6 ka BP.

### Permafrost

As shown during Pleistocene-Holocene transition few wetlands existed and the main source of methane could have been permafrost [8].

In northern Siberia during glaciation, mammoth steppe ecosystem existed [8], [23], [24]. In this ecosystem, water was often a limiting resource. Grass roots looking for water pierced the entire soil layer (down to the permafrost) where the temperature was never substantially higher than 0°C. Due to low decomposition, organic carbon accumulated not only on the surface but also in the deep active layer [24]. During glaciation on the planes of Siberia, massive loess strata (yedoma) accumulated. As dust accumulated on the soil surface, the lower soil horizons, together with organic remains, became incorporated into the permafrost. The carbon content in yedoma, despite low humus, is tens of kg/m^3^ and sometimes reaches 1 ton/m^2^ [25], [26].

During deglaciation the depth of the summer thaw increased and ice wedges began to melt. In such places, water filled depressions, and migrating thermokarst lakes appeared. During the course of deglaciation these lakes eroded half of the yedoma. At the present time, yedoma is only preserved on slopes or in the form of hills where new lakes cannot form. On lake bottoms, all yedoma became thawed and turned into many metres of silt. Part of the organic material was then transformed by methanogens into CH_4_. The diffusion of CH_4_ from sediment is/was very slow, and the main source is/was bubbling [27], [28].

Permafrost degradation is a very strong methane source (up to hundreds grams of CH_4_ per square meter per year) [27], [28]. On the one hand, the CH_4_ emitted from permafrost can be considered as a fossil since it is strongly depleted in ^14^C. (Note that the ^14^C content of atmospheric methane decreased during Pleistocene-Holocene transition [29].) On the other hand, the ^13^C and the deuterium content of methane from the Siberian permafrost is substantially lower than for all other sources (δ^13^CH_4_ = −73‰ (−58 to −99‰), δDCH_4_ = −400‰ (−338 to −479‰) [22], [27], [28]).

Permafrost methane emission is the only source that fulfils all of the following demands: it is the northern source; it is depleted in both deuterium and ^14^C; and its dynamics (Fig. 1H) are correlated with methane atmospheric dynamics in Pleistocene-Holocene transition (Fig. 1B).

During the LGM, the mammoth steppe resting on permafrost was the largest biome. It expanded from France to Canada and from the Arctic islands to China [8], [20]. In many regions of this biome, sediment deposition took place. As these sediments were accumulating they became incorporated into the permafrost. Such vast territories (besides the yedoma territory in the north of Siberia and Alaska) could be found in Europe, America, Siberia and China. Deposition of frozen sediments usually occurred with the accumulation of loess (area of 3*10^6^ km^2^, see Fig. 1 in ref. [8]) but also at the foot of slopes and in river valleys. On all of these territories thick strata, similar to yedoma, accumulated; similar in carbon content, but with fewer ice wedges [8], [24], [25]. In places where there was no notable sedimentation, soils were thin. At the beginning of the glaciation, when permafrost appeared, it was ∼1.6 m below the surface [24]. During glaciation, active layers decreased to ∼0.6–0.8. Therefore, the bottom portion of the soils became incorporated into the permafrost, even without sedimentation. Since these thin soils had developed for many thousands of years, they accumulated humus and the C concentration within them was two times higher than in yedoma [24]. During deglaciation, frozen soils of the mammoth steppe and “southern yedoma” thawed, not only underneath lakes (as in the north of Siberia), but ubiquitously; they lost their ice and passed the stage of anaerobic decomposition, which was followed by the drainage and aerobic decomposition stages – resulting in the loss of Pleistocene carbon and the transition to Holocene soils and loess [24], [25].

Methane emissions from northern Siberian permafrost have a very light isotopic signature. The light isotopic content of this CH_4_ is due to the following factors: (i) methane is released quickly and does not become fractionated by methanotrophs; (ii) methane production occurs very slowly at very low temperatures [22]; and (iii) methane production occurs in deep sediments through the decomposition of Pleistocene organic matter in Pleistocene water [30], [31]. The content of heavy isotopes declines in precipitation water as clouds move from south to north and from oceans to the interior of continents. Therefore, in northern Siberia the isotopic content of Pleistocene precipitation was very low, as indicated in the yedoma ice [30], [31]. Since the hydrogen used in photosynthesis and methanogenesis originates from precipitating water, the deuterium content in CH_4_ obtained from yedoma was also low. Our experimental methane isotope data was obtained for the coldest permafrost and for the most remote from the warm oceans sites in northern Siberia. The isotopic signature of methane from other “warmer” regions should be substantially heavier with isotopes. During the beginning of deglaciation, southern permafrost was largely degrading. For example, in Europe and China the signature of this source must have been substantially closer to the signature of boreal wetlands. With deglaciation, as permafrost degraded further on the north, the isotopic signature of the permafrost source must have turned lighter. The possible dynamics of this signature are shown in Table 1.

**Table 1:** The parameters values assumed in the model for A and B scenarios for all investigated time slices.

ka BP	18	15.5	14	12	11.2	10.6	9.2	8.4	7.4	5	3	1	0.2
**ATM, ppb**	370	470	600	500	730	730	730	660	650	580	610	675	730
**ATM δC13,‰**	−42.5	−44.3	−46.25	−44.9	−46	−46.6	−47	−47.25	−47.4	−48	−48.2	−48.3	−49.5
**ATM δD, ‰**	−80	−80	−93	−90	−108	−110	−110	−110	−110	−110	−105	−100	−115
**WT δC13, ‰**	−58	−58	−58	−58	−58	−58	−58	−58	−58	−58	−58	−58	−58
**WT δD, ‰**	−320	−320	−320	−320	−320	−320	−320	−320	−320	−320	−320	−320	−320
**WB δC13, ‰**	−64	−64	−64	−64	−64	−64	−64	−64	−64	−64	−64	−64	−64
**WB δD, ‰**	−327	−327	−327	−327	−327	−327	−327	−327	−327	−327	−327	−327	−327
**HV δC13, ‰**	−59	−59 (−61.5)	−59 (−63)	−59 (−63)	−59 (−65)	−59 (−65)	−59 (−65)	−59 (−65)	−59 (−65)	−59 (−65)	−59 (−65)	−59 (−65)	−59 (−65)
**HV δD, ‰**	−300	−300	−300	−300	−300	−300	−300	−300	−300	−300	−300	−300	−300
**BB δC13, ‰**	23	−23	−23	−23	−23	−23	−23	−23	−23	−23	−23	−23	−23
**BB δD, ‰**	−225	−225	−225	−225	−225	−225	−225	−225	−225	−225	−225	−225	−225
**GC δC13, ‰**	−60	−60	−60	−60	−60	−60	−60	−60	−60	−60	−60	−60	−60
**GC δD, ‰**	−190	−190	−190	−190	−190	−190	−190	−190	−190	−190	−190	−190	−190
**PF δC13, ‰**	−65.7	−65.7	−65.7	−65.7	−69.3	−69.8	−70.6	−71.3	−72	−72	−72	−72	−72
**PF δD, ‰**	−340	−340	−340	−340	−370	−374	−380	−387	−393	−393	−393	−393	−393

ka BP – thousands of years before present; ATM, ppb - atmospheric methane concentration in parts per billion units; δC13 and δD isotopic signatures of atmospheric methane or methane from specific sources. WT – tropical wetlands; WB – boreal wetlands; HV – herbivores methane source; BB –biomass burning methane source; GC – geological methane source; PF – permafrost methane source. If values differed between the A and B scenarios, the value for the second scenario is presented in brackets.

Unlike wetlands where annual emissions of carbon into the atmosphere are roughly equilibrated with carbon consumption with photosynthesis, degrading permafrost is only a source of carbon. The permafrost carbon reservoir was filled for tens of thousands of years within glacial steppes. Decomposition of this organic matter occurred in different conditions. The type of surface (thermokars lake, glacial lake, sea, wetland, or dry land) is not important for methanogenesis to occur during permafrost thawing. Organic materials decompose year round in deep sediments.

Local permafrost thawing, only in northern Siberia, could have released up to 26 Tg CH_4_/yr (Fig. 1H) [8]. We may expect that at least the same amounts were released from the total thawing of American, European, west and south Siberian, and Chinese permafrost, as well as permafrost from other regions, including Patagonian permafrost. However, in the southern hemisphere permafrost was rare, having mostly a northern source. Consequently, the permafrost source is enough to maintain the interhemispheric methane gradient [8]. The dynamics of permafrost emissions should reflect not the dynamics of temperatures in the northern hemisphere, but the dynamics of the above- and belowground deglaciation (lots of frozen soils were hidden under glaciers [32]). The dynamic of methane emissions from thawing European permafrost should be close to that of the north Siberian permafrost (Fig. 1H). After 6 ka BP, this source should have quickly depleted.

Modern permafrost emissions, connected with permafrost degradation under lakes and shallow seas, contribute approximately 10 Tg CH_4_/yr [8], [33]. It is unlikely that such a high emission could be recorded for the entire Holocene. Most likely, modern high permafrost emissions are connected with the climate warming and the active exploration of northern Siberia in the 20^th^ century. Before that, northern Siberia was poorly populated. In most ecosystems, the soil surface was covered with a thick layer of moss. Moss is a good heat insulator, and the depth of summer thaw under the moss in the yedoma region is only 20–40 cm. But if the moss layer is burned then the depth of the summer thaw (active layer) increases several fold. This often causes erosion and permafrost degradation [34]. This in turn leads to the creation of numerous ponds and thermokarst lakes [28]. In the 20^th^ century active exploration, mining operations and city and town construction took place. Annual precipitation on the yedoma territories is approximately 200 mm/yr (∼100 mm in summer) – it is a very dry region. According to our estimates, around half of all forests in the yedoma regions burned in the 20^th^ century. This disturbance caused increased active layer depth, rise of autumn-winter CO_2_ emissions from the soils and rise of seasonal amplitudes of atmospheric CO_2_ [35]. We suppose that must have increased the methane flux from the permafrost region as well.

### Herbivores

The LGM is a period of permafrost zone expansion and the accumulation of carbon in permafrost [25], [36]. ^14^C dating of thermokarst lake initiation dates indicated stable permafrost in the LGM (Fig. 1H). Therefore, we can infer that there was no methane emission from degrading permafrost during the LGM. As noted above, wetland and gas clathrates emissions were also minor. Therefore, some other sources must have constituted the main contribution during the LGM. From the list of well-known methane sources [12] only one big source is left – herbivores.

Lets make some investigation and rough estimates on whether could this source have played important role the late Pleistocene? Modern methane emissions from wild herbivores are approximately 2–6 Tg/yr [37], but herbivores were more abundant in the past. During the last deglaciation, tens of megafauna species became extinct, and a hypothesis even exists which states that during the Younger Dryas CH_4_ decline was caused by a decline in megafauna in North America [38].

Current animal populations in grasslands are mostly limited by human regulations, while in the Pleistocene, before man invented reliable hunting tools and learned how to hunt all animal species, herbivore biomass was limited by the forage available for animals. During the winter/dry season, under high animal diversity, everything that grew during the summer/rainy season was eaten [23], [34], [39], [40]. Everything uneaten by bulls and horses was eaten by omnivorous goats. If available food resources were left unused on the pastures in the spring, mortality related to starvation was rare and the animal population grew in the following summer, and by the following year all the available forage was eaten. For all wild ruminant animals (e.g., bulls, antelope, deer, goats, etc.), methane production is 28.3 kg per ton of forage, or ∼100 kg/yr per ton of animal weight [37]. For non-ruminant animals (e.g., proboscideans, horses, pigs, etc.), CH_4_ production is ∼4.5 times smaller [37].

Currently, the area of pasture and cropland on Earth is 35.3×10^6^ km^2^. Harvests from these territories maintain a “megafauna” population of ∼1.2 Pg (34 tons per km^2^ of pastures), one-third of which is human biomass and two-thirds of which comprises domestic animals, mainly ruminants, that produce ∼90 Tg CH_4_/yr [37]. During the LGM, forested areas were ten times smaller than during the Holocene, and the area of grass–herb dominated ecosystems, reached 70×10^6^ km^2^ [19]. I.e. pasture area was twice of modern pastures and agricultural fields.

Vegetative periods on wild pastures are longer than on tilled fields. On pastures the entire biomass is utilized, and not only seeds and fruits. In the LGM there were no “technical” plant species, like cotton. On pasture, nutrients are not removed from the fields with the harvest but quickly and uniformly returned to the bio cycle. Taking all this into account, we can suppose that herbivore methane emissions in the LGM could be no less than the modern 90 Tg/yr.

We obtained more precise estimates of animal densities and their methane emissions for northern Siberia, where, because of high rates of yedoma accumulation, high numbers of late Pleistocene animal skeletons and bones are preserved [34], [40]. We did estimates with different methods and for different regions. As a result, it was ascertained that in the late Pleistocene (over the 40 ka period) on the plains of the Siberian north, on each square kilometre approximately 1,000 of mammoths, 20,000 bison, 30,000 horses and 80,000 reindeer had died. From that it was calculated that, on average, for every moment of time on each square kilometre one mammoth, five bison, 7.5 horses and 15 reindeer roamed. Accounting for other more rare animals, the biomass of herbivores equalled 10.5 ton/km^2^ [34], [40]. Half of that biomass belonged to ruminant bison and reindeer with the rest being non-ruminant mammoths and horses [34], [40].To maintain this biomass, approximately 100 tons of dry biomass per annum was supposed to be consumed from each square kilometre.

Even today such a high animal density in high- and mid-lattitudes can be observed. The modern density of semi-wild Yakutian horses on the highly productive meadows of northern Siberia –30 horses/km^2^ [34], [40], which roughly corresponds with our calculations. The same density can be observed in the pastures of the” Pleistocene Park” in the Kolyma river lowland [34], [40]. In the “Oostvaardersplassen” park in the northern Netherlands, animal density is not regulated by man, but determined only by pasture productivity. There animal density is stable around 100 hoofed animals per square kilometre [41].

In the LGM, animal density in the north was substantially lower than on average for the period of the late Pleistocene [34], [40]. Therefore, we can accept that on each square kilometre of plane land (yedoma) pasture in LGM only 50–70 tons of forage were consumed. North of Siberia is the coldest and driest part of the mammoth steppe biome and this biome in turn comprised the most severe steppes in the world. In warmer and wetter climates, productivity (and consequently animal density) was probably higher. If it is assumed that, on average, on the planet herbivores consumed 100–140 tons/km^2^, the area of pastures was 70*10^6^ km^2^ and ruminant animals constituted half of all the big herbivores, then global methane herbivore emissions could potentially reach 120–170 Tg/yr.

One of the features of the herbivore source is a quick and strong response to changes in precipitation. If in arid climates precipitation increased, then the productivity of grasses and herbs would increase in the same year, and in 1–2 years the number of ungulate and herbivore methane emission would increase respectively. This most likely occurred in the beginning of the Bølling warming (similarly, increase in herbivore density and herbivore methane productions should have been observed during all events of increased precipitation over the course of the Pleistocene). Activation of other sources, such as wetlands, or permafrost thawing appeared later. Eventually forest expansion caused pasture areas to decline and the herbivore methane flux have probably decreases. Additionally, the warming favoured the expansion of man to the north and to America. Due to increased hunting pressure, a number of herbivores declined at that time [34], [40].

### Biomass Burning

A direct proxy of fire intensity dynamics is the amount of charcoal in stratigraphic columns, but such data are rather sparse. Relatively reliable data are published only for the territory of the USA [42]. They showed a strong rise in fire activity in the Bølling-Allerod and Preboreal [42]. For north-eastern USA, data are obtained simultaneously with data on the abundance of dung fungus spores, which characterize the amounts of dung in the sediments and consequently the animal density. These data indicated that during the LGM, in north-eastern USA fires were rare, herbivores consumed everything, and there was nothing to burn [43]. After herbivore disappearance, fires became “persistent” [43]. Similar dynamics have been recorded for various regions ([39] and references therein).

## Methods. A Methane Isotope Model

During methane oxidation, isotopic fractionation causes the isotopic content of atmospheric CH_4_ to differ from the isotopic content of the weight averaged CH_4_ source through the values of KIE (kinetic isotopic effects).

The KIE coefficients of δ^13^CH_4_ and δDCH_4_ are only moderately dependent on temperature and methane lifetime, and can be considered to be constants, allowing one to reconstruct the dynamics of the weighted average CH_4_ source’s isotopic content by knowing the dynamics of the isotopic content of atmospheric methane [6], [11], [12].

To reconstruct the dynamics of the main sources of CH_4_, for every time slice we investigated we used the following set of equations [6], [11], [12]:
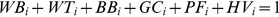
(1)




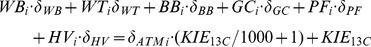
(2)




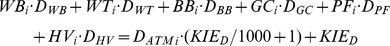
(3)where WB, WT, BB, GC, PF, HV are CH_4_ sources from respectively boreal wetlands, tropical wetlands, biomass burning, gas clathrates, permafrost and herbivores, units are the ratio of total global emission;D and δ are the isotopic signatures of all of the corresponding sources, in ‰ (presented in Table 1 and fig. 2 [12]);KIE_13C_ and KIE_D_ are the coefficients of atmospheric isotopic fractionation, in ‰.i, number of time slice (see column labels in the Table 1 for details on investigated time slices).

These formulas show the relationship between the isotopic signatures of methane sources and the global atmosphere. As input parameters we have the isotopic record from the Greenland ice cores [11], [12], [13] (blue line in Fig. 2 and Table 1)(i.e., we know the isotopic signatures of the atmosphere above Greenland), but for our calculation globally averaged values are necessary. Therefore, as a first step we need to find a connection between the global atmosphere and the atmosphere above Greenland. In the southern hemisphere, non-tropical sources are minor. Therefore, the situation where the entire methane atmosphere becomes isotopically lighter, while becoming heavier above Greenland during the same period of time, is not possible. The dynamics of Greenland methane should be roughly parallel with the global methane dynamics. The δ^13^CH_4_ from Greenland is different, by less than 1‰, from the δ^13^CH_4_ of Antarctica and the averaged atmosphere, and remained the same for the period of time spanning from deglaciation to 1 ka BP [13]. In Figure 1, one can see that the values of δ^13^CH_4_, obtained for the Younger Dryas through the Preboreal periods in Greenland (GISP 2) and Antarctica (EDML), are similar. We assumed that during the LGM the difference also remained small.

The δDCH_4_ of the atmosphere above Greenland 1 ka BP, differed from Antarctic values by ∼12‰, and from averaged atmospheric values by ∼8‰ [13]. LGM emissions of isotopically light methane from boreal wetlands and permafrost were near zero and the interhemispheric methane gradient was low. Herbivores and biomass burning doesn’t influence inter-hemispheric methane gradient. Therefore, the LGM interhemispheric gradient of methane isotopes should also be low. As a first approximation, we can assume that interhemishperic gradients of atmospheric methane concentrations and the isotopic contents are proportional to each other. We assumed that the δDCH_4_ from Greenland differed by 8‰ from the averaged atmospheric values for the Holocene, by 4‰ for the Younger Dryas (12 ka BP), by 6‰ for the Bølling-Allerod period (14 ka BP), and by 2‰ for the LGM and 15.5 ka BP.

**Figure 3:**
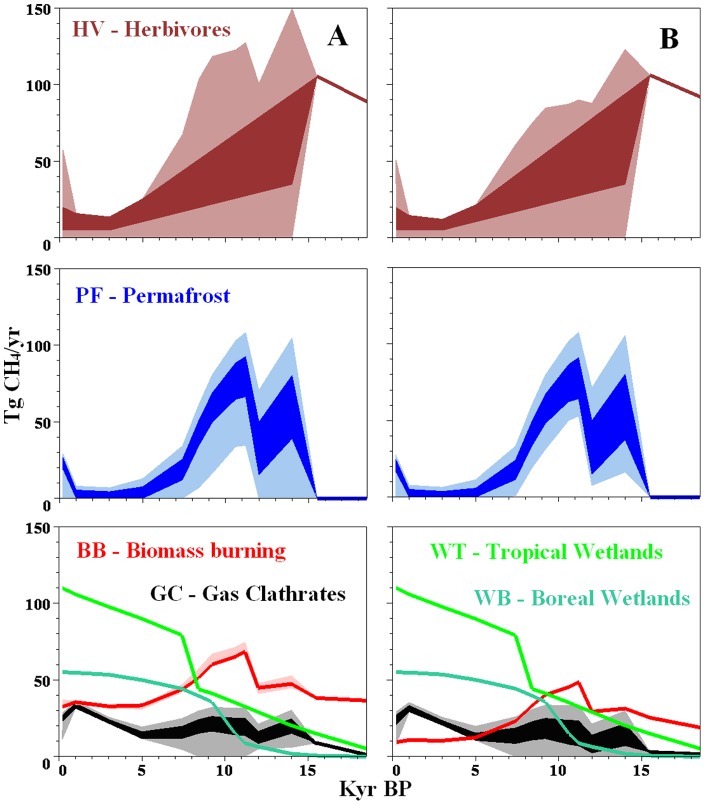
Model estimated ranges of the possible values for methane emissions for herbivore, permafrost, biomass burning and gas clathrates for time slices from the LGM to the pre-industrial. Dark shaded areas represent calculated ranges obtained following the additional restriction placed on herbivore and permafrost emissions. Wetland dynamics are provided as generalized graphs in Figs. 1F and 1G. A – scenario calculated for KIEs −6.8‰ and −218‰ [12]; B- scenario calculated for KIEs −10.8‰ and −227‰ [6].

As a result, we ascertained the dynamic of isotopic signatures of global atmospheric methane. During atmospheric methane oxidation isotopic fractionation takes place, therefore methane in the atmosphere is much heavier than the source methane. This fractionation is described by parameters of KIE for both deuterium and ^13^C. The *Fischer et al.* [12] model utilized KIE values of −6.8‰ for δ^13^CH_4_ and −218‰ for deuterium. We utilized this pair of KIEs in our first scenario (Fig. 3A).

Inclusion of both isotopic dynamic of atmospheric CH_4_ and KIEs allows the calculation of the right hand sides of Equations 2 and 3. It is the dynamic of the isotopic content of the weighted average global source (purple line in Fig. 2).

We have three equations and six sources (strengths of six sources of methane) for each time slice. But for the LGM time slice we know that the emissions from permafrost, tropical and boreal wetlands are close to zero. Therefore, for the LGM we can solve the equations directly: herbivore emissions constituted 69% of global emissions, biomass burning 39% and gas clathrates emission 2%. Without knowing the lifetime of methane in the atmosphere in the LGM it is impossible to reconstruct the size of the total global methane emission. But if we were to accept that total herbivore methane emissions in the LGM were 100 Tg/yr, then global emissions were 145 Tg/yr, and accounting for the methane content in the atmosphere in the LGM (∼1000 Tg CH4), the life of methane was ∼6.9 years.

The third step is to set values of tropical and boreal wetland methane emissions for all time slices. The wetland succession model we created based on peatland initiation dates (thin lines in Figs. 1D and 1E) allow the detection of the relative dynamic of these sources. Recent estimates for modern tropical and boreal wetland emissions are 110 and 55 Tg CH_4_/yr [44]. Accepting these values for the pre-industrial time slice allows the reconstruction of the wetland methane source from the LGM to the present for all time slices. In Equation 1 all source units are the ratios of total global methane flux. To estimate the strength of wetlands sources not in Tg/yr units but in the ratios of total, for every time slice the methane lifetime should be considered.

Lifetime is an unknown and poorly constrained parameter in our model [6]. But solving the system of equations for numerous time slices, from the LGM to the present, and having reasonable (or any) solutions is possible only in narrow range of input parameters, including lifetime.

Accepting tropical and boreal wetland emissions of 110 and 55 Tg CH_4_/yr [44] for the pre-industrial time slice, we automatically set the upper limit of methane lifetime values. If we were to accept a lifetime of more than 8.6 years then late Holocene solutions of Equations 1–3 would be lost. In turn, if the lifetime is shorter than 7.6 years, herbivore emissions during the late Holocene would exceed reasonable values (25 Tg/yr). At that time, wild herbivores were already rare and domestic animals were still few. Therefore, for the first scenario, we assumed 8 years for all time slices. It is likely that this value is correct for the entire Holocene. For Pleistocene-Holocene transition and the LGM it could be different. However wetland emissions were minor at that time; therefore changes in lifetime have little effect on the relative strength of other sources. If required, we can change the lifetime of methane during Pleistocene-Holocene transition and in the LGM, which would consequently change the strength of all sources in Figure 3 at that time. If we change the lifetime of methane in the atmosphere, it would change the strength of all sources in Tg/yr units, but emissions in units of the ratio from global emissions would stay intact.

After accepting boreal and tropical wetland emissions values for every time slice as ratios of global methane emission we obtain a system with three equations (Eq. 1–3) and four unknowns (biomass burning, gas clathrates, permafrost and herbivores). This does not allow us to calculate the dynamic of the source, but it allows us to estimate possible ranges of strengths of these sources for every time slice (Fig. 3). True estimates could only be made for the LGM and 15.5 ka BP time slices, where permafrost source was taken as 0.

The model’s solution can be presented visually. For each time slice, in Figure 2, global emissions should be divided between individual sources, in the way “centre of gravity” would coincide with the position of the weighted average source (knots in the purple line). Model reactions to different changes can also be evaluated visually. “Remote” sources – biomass burning, gas clathrates and permafrost – have a “long lever”, therefore the shift in strength by a few Tg/yr of these sources, noticeably shifts the “centre of gravity”. On the other hand, an error in the isotopic signature of these sources does not influence solutions very much. For example, a few per mil shift in the biomass burning source signature in Figure 2 would still leave this source far to the left from the “centre of gravity”. Tropical wetlands and herbivores have a “short lever” therefore the change in strength by a few Tg/yr of these sources is not noticeable in the solutions, while an error in the isotopic signatures (position of these sources in Fig. 2), can substantially bias our solutions.

Modelling results are the most sensitive to changes in KIEs. These two parameters define the position of the purple line on Figure 2. If this line were slightly shifted towards the biomass burning, then the ratio of this source in global emissions would increase (and the ratio of other sources would consequently decrease). If the purple line were shifted to the bottom right, then the ratio of gas clathrates would increase. But if the purple line were further shifted towards the gas clathrates, then for fixed wetland emissions and lifetimes, the solutions in our model would be lost – the dynamic of methane isotopes in the atmosphere (Figs. 1B and 1C) does not allow substantial gas clathrate sources, regardless of the assumed KIE parameters.

In the available literature, KIE coefficients are broadly varied [6]. For δ^13^CH_4_ an estimate of −6.8‰ exists, but a more recent value of −10.8‰ also exists [6]. The KIE for deuterium is −218+50‰ [5]. The *Fischer et al.* [12] model utilized KIE values of −6.8‰ and −218‰. We utilized this pair of KIEs in our first scenario as well (Fig. 3A). In this scenario, we had very high biomass burning methane emissions in the LGM (Fig. 3A). But this contradicts the high density of animals at that time – actively grazed pastures do not supply much organic material for fires [43].

For the second scenario (Fig. 3B) we used KIEs of −10.8‰ and −227‰, shifting the purple line in Figure 2 to the upper right. For these KIEs, solutions existed for all time slices. Moreover, we obtained solutions for the modern methane budget as well, but in order to do so had to make “permafrost corrections” in the modern budget. The ratio of fossil sources (coal and gas) in the modern budget is calculated through the ^14^CH_4_ budget [6]. The ^14^C age of methane in the atmosphere allows the calculation of the ratio of all sources that are fossil sources. Permafrost is also a fossil source (strongly depleted in ^14^C) but it was not considered in reconstructions of the modern methane budget. Unlike gas and coal heavy with isotopes [6], permafrost is very light. For the modern budget it was necessary to add permafrost, and correspondingly reduce the ratio of other fossil sources. The current permafrost source is relatively small (∼10 Tg/yr) but it has very distinct deuterium and ^13^C signals compared to coal and gas; therefore taking even a small weight from one long “lever” and placing it with the opposite long “lever” (Fig. 2) substantially changes the isotopic content of global emissions. In the modern budget several additional anthropogenic sources are considered. By adding 10 Tg/yr emissions from permafrost to it, for KIEs of −10.8‰ and −227‰ we have managed to obtain values of all modern sources in the frames of their known ranges. Therefore, we saw no reason not to use KIE values of −10.8‰ and −227‰ for paleo reconstructions.

Since herbivores are a strong source with an isotopic signature close to the weighted average source signature, the parameter with the second strongest impact on our model results was the isotopic signature of this source. In the first scenario we used δ^13^C for herbivores, as in the Fischer model, for all time slices. *Fischer et al.* used a δ^13^C for HV of −59‰_,_ as for modern animals, with a high proportion of C_4_ plants (e.g. corn etc.) in the diet [6], [45]. This value is likely to be true for the LGM also, since, during low atmospheric CO_2,_ the ratio of C_4_ plants increased and the δ^13^C in C_3_ plants decreased by 2‰ [46]. However, for other time slices, CO_2_ concentrations were higher. For Pleistocene-Holocene transition we used the herbivore signature in compliance with atmospheric CO_2_ concentrations [46]. So, in the second scenario for the LGM, we assumed −59‰ (as in Fischer model); for the time slice of 15.5 ka BP we used −61.5‰, for the Bølling-Allerod and Younger Dryas we used −63‰, and for the Preboreal and Holocene we used 65‰. In calculating the modern budget we have utilized the same value as Fischer, −59‰. Detailed data of all assumed isotopic signatures of all sources can be found in Table 1.

For the second scenario, using the same restrictions as for the first, the lifetime was determined to be 9 years.

## Results

Modelling results for both scenarios are presented in Fig. 3, we see that for the herbivore and permafrost ranges in our model an additional correction was required. The model returned clearly overestimated maximum values for these parameters. During deglaciation, permafrost emissions could not be substantially greater than the northern flux obtained by the interhemispheric gradient. During the Holocene, forested areas increased and pasture areas decreased. Additionally, numerous herbivore species became extinct. If current herbivore emissions are 90 Tg/yr, then 200 years ago, when human and domestic animal populations were much lower, emissions could not be as high as 50 Tg/yr. Any corrections for any ranges can easily be made using Figure 3. Since the equations in our model are linear, the values for all of the calculated sources are interconnected. The herbivore source is located inside the triangle of permafrost, gas clathrates and biomass burning (Fig. 2). Therefore, the herbivore source maximum corresponds to the minimum for permafrost, gas clathrates and biomass burning, and vice versa. If, for any time slice in Figure 3, we, for example, restrict the range of permafrost by one-third from the top, then the gas clathrates and biomass burning ranges would be restricted by one-third from the top as well, and the herbivore source range would also be restricted by one-third, but from the bottom. If we accept a middle value from the range of one specific source then the values of the other three sources would also be located in the middle of their ranges.

For our work, we only made the most obvious and least controversial correction (see paragraph above) – we restricted the ranges of herbivores and permafrost from the top. However, reducing the range of herbivores from the top, restricted the permafrost, gas clathrates and biomass burning source ranges from the bottom; while restricting the range of permafrost sources from the top led to restricted range of biomass burning and gas clathrates sources from the top and restricted herbivore source ranges from the bottom. As a result we obtained relatively narrow ranges for all sources (the dark shaded sector in Fig. 3).

Testing our model we changed all of the initial parameters over a wide range, and implemented additional sources. In all cases, if solutions existed, the dynamics of the sources were similar to the results in Figure 3. We are not confident that 110 and 55 Tg CH4/yr are correct estimates, and tested our model for other values of modern wetlands emissions. This required accepting different lifetimes of methane. However, the main results of our work stayed intact.

All modelling was done on Maple v.10.0 software.

**Biomass burning** emissions in all scenarios had a very narrow range (Fig. 3), and responded strongly to variations in initial parameters but in all cases strongly increased from 15.5 to 12 ka BP. As was noted earlier, similar dynamics for fires were reconstructed using charcoal records [42].

**Gas clathrate** emissions, as we predicted, were low for all of the scenarios. For the end of the Holocene, for all scenarios, we determined a clear peak of gas clathrates (as suggested by Sowers, [13]).

Earlier we mentioned that methane from the bottoms of deep seas could not penetrate into the atmosphere therefore GC emissions during the Holocene (Fig. 3) could be connected with the land [47] or with shallow Siberian seas [33]. During the LGM, their bottoms were land and were armoured with a gas-proof thick frozen layer. Only when these layers thawed could trapped gas escape. Soils of the mammoth steppes are relatively thin (10–60 m) and thaw quickly. Gas clathrates are located deep, and, in order to thaw hundreds of metres of permafrost, thousands of years are required. Therefore, as hundreds of metres of permafrost degraded in the second part of the Holocene, gas clathrates emissions could grew (Fig. 3).

In all scenarios global **permafrost** emissions closely followed atmospheric methane dynamics and permafrost degradation in the Siberian north (Fig. 1H). If emissions from boreal wetlands and permafrost were combined then the dynamic of this united northern source would be compiled with the interhemispheric CH_4_ gradient. By integrating the permafrost emission data (Fig. 3), we found that the source released ∼300–550 Pg CH_4_ (225–412 Pg C) to the atmosphere from 15 to 6 ka BP. During the Holocene, permafrost sources quickly diminished, while boreal wetlands emissions grew. This explains why the interhemispheric gradient was relatively stable in the Holocene.

In pre-industrial times (200–400 years ago), a sharp decline in the deuterium content in atmospheric methane took place (Fig. 1C). The model was sensitive to this decline and showed that this decline was caused by the activation of permafrost emissions (Fig. 3). Increased degradation of permafrost at that time made sense; at that time, active colonization (exploration) of Siberia and Alaska was underway and in the north many settlements and towns, connected with a transport network, appeared. Sable trapping became an activity not only for colonizers, but also for indigenous people for the first time [48]. With colonization, fire intensification must have taken place and permafrost degradation must have been activated.

**Herbivore** emissions during the Pleistocene, for a methane lifetime of 8–9 years, were 90–100 Tg/yr. If we were to accept a shorter lifetime, then herbivore emissions could be even higher. These estimates are close to our estimates obtained via global forage productivity (see Herbivore section in Background). Herbivore emissions until 15.5 ka BP were close to the capability of world pastures, decreased during the Holocene, and reached a minimum during the late Holocene. Wild animals were already rare, and domestic animals were still found in small numbers.

## Conclusions

Today wetlands are main natural source of methane and it is natural and easiest to suppose that it was same in the past. However we haven’t found any proofs of this hypothesis. The main conclusion of our work is that permafrost and herbivores in the past were not only important but also the main methane sources. The main fact from which these two conclusions follow is that in the LGM and during Pleistocene-Holocene transition there is little evidence of peat or anaerobic conditions in the soils. From that it follows that, at that time, wetland methane emissions were at least several times weaker then today. Therefore, some other sources were major at that time. Since, besides boreal wetlands, we know of only one northern methane source (permafrost), we can conclude that permafrost was responsible for the interhemispheric gradient dynamic in deglaciation. In the LGM, permafrost was stable and the main methane source (in the absence of wetlands) could only be herbivores.

These results were obtained independently from isotopic model. The model in turn also depends on data about the lack of wetlands in the LGM and during Pleistocene-Holocene transition, but it is independent of data about the interhemispheric gradient.

The isotopic model we used is simple and broadly accepted [6], [11], [12]. We have only added the unknown permafrost source, and utilized the most obvious restrictions – herbivore methane emissions in the Holocene could not be higher than in the LGM and in the end of the Holocene could not exceed 25 Tg/yr; permafrost emissions could not be higher than indicated by the interhemispheric gradient.

The model has allowed the dynamic of all sources to be reconstructed in more detail. Additionally, noting that only the dynamic was reliably reconstructed, absolute values of the strength of each source have been estimated very approximately. Our estimates rely on modern estimates of wetland emissions. If true values differ from the assumed, then to solve the model equations different methane lifetimes should be taken. Our model estimates are rough, we did not consider some of the sources; for example, in the late Holocene some of the wetlands were transformed into rice paddies [9].

We know the values of the isotopic signatures of all sources and the KIE, possibly with substantial errors. These values can be corrected in the future. But if the position of each source in Figure 2 relative to each other will not change, then with new corrected values either solution of the model will not exist (wrong incoming values) or the model will show that herbivores were the main source in the LGM and permafrost during deglaciation.

## Discussion

The cold and well aerated soils of mammoth steppe are unlikely to have produced methane in the Pleistocene, and are likely to have accumulated carbon [24], [34], [40]. This would certainly have affected concentrations of greenhouse gases in the atmosphere. Even modern permafrost soils are rich in carbon and are estimated to contain 1672 Pg C [49] globally. In the LGM, the mammoth steppe was the biggest biome and permafrost covered a much larger area. Estimating carbon storage in permafrost during the LGM has been a significant challenge [24], however, we can now make rough estimates using methane emissions from permafrost sources estimated in this study. Integrating the curve in figure 3, we estimate that permafrost emitted 400 Pg CH4 (300 Pg C) into the atmosphere during the deglaciation. What was the total carbon loss from the northern soils over that time period? Even in fully anaerobic conditions only part of the stored organic (28±12%) can be transformed into methane [50]. When thick (tens of meters) frozen ice-rich soils (yedoma and its southern analogues) thaw they are initially anaerobic, however, as permafrost degradation continues, underlying gravel and sand thaws and water begins to drain from shallower soil layers. These soils become aerobic and carbon is transformed into the CO2. However on most territories occupied by the mammoth steppe, soils (both including active layer and soils incorporated into the permafrost) were shallow (less than 2–3 meters deep), and carbon stored in these soils was mostly decomposed under aerobic conditions when the climate warmed. Therefore, if we accept that 15% of permafrost carbon loss was transformed into methane, we can estimate that permafrost soils lost 2000 Pg C during deglaciation (15–6 ka BP).

As current climate warming proceeds, permafrost temperatures are increasing and widespread permafrost degradation is projected. Currently, permafrost soils contain 1672 Pg of carbon [49]. What portion of this carbon will be transformed into methane as these soils begin to thaw? We assumed 15% during deglaciation. We could not take smaller value, since that would have returned an unrealistically big estimate of the permafrost carbon reservoir in the Pleistocene [24]. In modern permafrost, a higher proportion of carbon has already been thawed in the Holocene and then refrozen. It is unlikely that at future thawing such soils would produce lots of methane because much of the easily-decomposed forms of carbon have already been processed. We estimate that no more than 10% of organic carbon in modern permafrost is likely to be transformed into methane as the climate continues to warm. Thus, we estimate that modern permafrost pool has a potential to release 167 Pg C in the form of methane. If future permafrost degradation (in contrast to the Pleistocene-Holocene transition) is rapid and a quarter of this methane is released within 200 years, average methane emissions will be ∼250 Tg/yr. This value will make degrading permafrost world biggest methane source.

If our calculations of animal densities in the Pleistocene are correct (or at least close), it appears that wild nature solely on recycled resources managed to sustainably maintain biomass higher than the entire biomass of modern civilization (humans and all domestic animals) which is supported only by burning enormous amounts of non-refundable resources. This results draws attention to the ineffectiveness of current human management of the precious, finite resources available on our planet.
